# Establishment and characterization of a *Wolbachia* (*w*AlbB)-infected *Aedes aegypt*i line (Tw-Kao) for dengue control

**DOI:** 10.1371/journal.pntd.0014222

**Published:** 2026-05-04

**Authors:** Hui-Ying Yu, Bo-Yu Chen, Ying-Tsong Chen, Wei-Kang Huang, Cheng-Hao Yang, Yung-Chieh Wu, Mei-Hsiu Wan, Wen-Sheng Yeh, Feng-Guang Goh, Xinjun Hou, Pui-Kuan Toh, Sui-Hann Ng, Yu Cai, Jian-Chiuan Li, Chun-Hong Chen, Wei-Liang Liu

**Affiliations:** 1 Institutes of Molecular and Cellular Biology, National Taiwan University, Taipei, Taiwan; 2 National Mosquito-Borne Diseases Control Research Center, National Health Research Institutes, Miaoli, Taiwan; 3 Graduate Institute of Genomics and Bioinformatics, National Chung Hsing University, Taichung, Taiwan; 4 Institute of Molecular and Genomic Medicine, National Health Research Institutes, Miaoli, Taiwan; 5 Temasek Life Sciences Laboratory, 1 Research Link, National University of Singapore, Singapore, Singapore; 6 Department of Biological Sciences, National University of Singapore, Singapore, Singapore; 7 National Institute of Infectious Diseases and Vaccinology, National Health Research Institutes, Miaoli, Taiwan; Instituto Oswaldo Cruz, BRAZIL

## Abstract

Global trade and climate change are driving the geographic expansion of dengue vectors, contributing to the global spread of dengue. Conventional vector control measures have proven insufficient to prevent substantial disease burdens, highlighting the need for innovative and sustainable strategies. The release of *Wolbachia*-infected mosquitoes offers a promising alternative for dengue suppression. Here, we developed a locally derived *Ae. aegypti* line carrying the *w*AlbB strain (*w*AlbB-Tw-Kao) and systematically evaluated its fitness, viral interference, and potential for vector population control. The strain was generated through embryonic microinjection of cytoplasm containing the intact *w*AlbB endosymbiont from field-collected *Ae. albopictus* in Kaohsiung, Taiwan, resulting in a stably infected mosquito line with 100% maternal transmission. Whole-genome sequencing confirmed a high similarity to the reference *w*AlbB genome. Cross-mating experiments demonstrated complete cytoplasmic incompatibility (CI, 0% egg hatch) when *w*AlbB-Tw-Kao males were mated with uninfected females. Antiviral assays against dengue virus serotype 2 (DENV-2) and Zika virus showed significant reductions in viral titers in the midgut, salivary glands, and saliva. In cage experiments, increasing release ratios of *w*AlbB-Tw-Kao males led to significant suppression of wild-type populations, achieving up to approximately 90% reduction in egg hatch. These findings demonstrate the successful development of a locally derived *w*AlbB-infected *Ae. aegypti* line with strong CI, stable maternal transmission, and effective DENV and ZIKV blocking. These properties provide a foundation for future field-relevant evaluation under both suppression and replacement deployment frameworks.

## Author summary

Dengue is spreading rapidly worldwide, and conventional mosquito control methods alone are no longer sufficient to prevent outbreaks. A promising new approach uses *Wolbachia*, a bacterium that can reduce mosquito fertility and block virus transmission. In this study, we developed a locally adapted *Ae. aegypti* mosquito line carrying the *w*AlbB strain of *Wolbachia* (named *w*AlbB-Tw-Kao) in Kaohsiung, Taiwan. The *w*AlbB-Tw-Kao *Ae. aegypti* mosquito line was stably inherited by all offspring and induced complete cytoplasmic incompatibility (CI), resulting in embryonic lethality when infected males mated with uninfected females. Laboratory assays demonstrated significant inhibition of dengue and Zika virus replication in mosquitoes. When released in cage trials, *w*AlbB-Tw-Kao males effectively suppressed wild-type mosquito populations. These results demonstrate that *w*AlbB-Tw-Kao is a promising candidate for sustainable dengue control in Taiwan and other dengue-endemic areas.

## Introduction

Dengue fever is an acute viral illness caused by the dengue virus (DENV), primarily transmitted through the bites of *Aedes* (*Ae.*) *aegypti* and *Ae. albopictus* [1]. The disease is now endemic in 129 countries worldwide, with more than 5 million reported cases annually, posing a significant threat to global public health [2,3]. Currently, there is no effective antiviral treatment for dengue, and clinical management relies mainly on supportive care. Although vaccines have been developed, safety in seronegative individuals remains a concern as vaccination may increase the risk of severe disease. Their overall effectiveness in public health interventions is still debated [4,5]. Consequently, dengue control efforts continue to focus on mosquito vector management, primarily through source reduction and insecticide spraying. However, the growing prevalence of insecticide resistance has diminished the efficacy of these conventional methods, creating a major obstacle to global dengue control [6,7]. In response, many countries have recently invested in alternative biological control strategies, such as the release of *Wolbachia*-infected mosquitoes and genetically modified mosquitoes [8–11]. These innovative approaches reduce reliance on chemical insecticides and the risk of resistance, making them promising components of future integrated vector management programs. Importantly, *Wolbachia*-based strategies can be implemented either as population suppression approaches, which reduce mosquito density through cytoplasmic incompatibility, or as population replacement strategies, which introduce virus-resistant mosquitoes into wild populations to interrupt transmission.

*Wolbachia* is a maternally inherited, obligate intracellular symbiotic bacterium belonging to the order Rickettsiales within the class α-proteobacteria. It is estimated that approximately 65% of insect species harbor *Wolbachia*, making it the most widespread intracellular symbiont known to date [12]. *Wolbachia* manipulates host reproduction and evolution through various mechanisms, including lifespan modulation, feminization, parthenogenesis, male killing, and the most commonly observed phenomenon—cytoplasmic incompatibility (CI) [13–16]. CI occurs when *Wolbachia*-infected males mate with uninfected females, resulting in embryo lethality and unsuccessful hatching. This selective advantage facilitates the spread of infected individuals within a population, providing a foundation for vector control strategies.

Current vector control strategies utilizing *Wolbachia* can be broadly categorized into two approaches: population suppression and population replacement [17,18]. The population suppression strategy exploits *Wolbachia*-induced CI, whereby the release of *Wolbachia*-infected male mosquitoes into the field results in embryonic lethality when they mate with uninfected wild-type (WT) females [19,20]. Multiple *Wolbachia* strains have been applied or evaluated for population suppression in different mosquito species, including *Ae. albopictus* and *Ae. polynesiensis*. This strategy, known as the incompatible insect technique (IIT), effectively reduces mosquito population density and limits the transmission potential of vector-borne diseases. Because only males are released—and males do not bite or transmit pathogens—this approach minimizes the risk of increased nuisance biting or disease transmission. However, as sex-sorting procedures are not perfectly efficient, accidental release of infected females may occur, underscoring the importance of stringent quality control and monitoring to prevent unintended *Wolbachia* establishment in wild populations [21,22]. IIT has been successfully implemented in China, Singapore, and the United States (e.g., Verily’s Debug project), demonstrating substantial suppression of *Ae. aegypti* populations across multiple settings. A corresponding reduction in dengue incidence has been documented in Singapore, whereas epidemiological outcomes were not assessed in the China and United States studies [18,19,23].

In contrast, the population replacement strategy involves the release of both male and female mosquitoes carrying *Wolbachia*, enabling vertical transmission of the symbiont and gradual fixation in wild mosquito populations [21,24,25]. Certain *Wolbachia* strains, including *w*Mel and *w*AlbB, have been shown to inhibit the replication of RNA viruses in *Ae. aegypti*, thereby limiting viral dissemination within the mosquito and reducing transmission risk to humans [26–29]. The magnitude of antiviral activity can vary among *Wolbachia* strains and host genetic backgrounds. In addition, lifespan reduction has been reported for certain *Wolbachia* strains, such as *w*MelPop, which can limit virus transmission by reducing the probability that infected mosquitoes survive the intrinsic incubation period [30]. However, this phenotype is not consistently observed across all *Wolbachia* strain–host combinations. Large-scale field deployments of *Wolbachia*-infected mosquitoes have been conducted in multiple countries, including Australia, Brazil, Colombia, Indonesia, and Malaysia, where stable establishment and long-term persistence of infected mosquito populations have been achieved. Significant reductions in dengue incidence have been documented in several settings, including Australia, Indonesia, and Malaysia, following deployment of *w*Mel or *w*AlbB strains [25,31–36]. Taken together, *Wolbachia*-based strategies represent a promising and sustainable innovation in mosquito vector control. By either suppressing vector populations or disrupting viral transmission pathways, these approaches offer a scientifically robust and environmentally safe alternative to conventional chemical-based interventions in the face of growing global arboviral threats.

In Taiwan, not stably naturally *Wolbachia*-infected *Ae. aegypti* line suitable for vector control is currently available. Although low-prevalence *Wolbachia* detections have been reported in field-collected *Ae. aegypti* [37], these do not constitute an operationally deployable, stably inherited infection. To bridge this gap, the *w*AlbB strain was isolated from wild *Ae. albopictus* populations collected in Kaohsiung, southern Taiwan. Using embryonic microinjection techniques, *w*AlbB was successfully transinfected into a local *Ae. aegypti* line, resulting in the establishment of a stable *Wolbachia*-infected Taiwanese strain. Following successful line establishment, we conducted a series of evaluations to assess its biological characteristics and phenotypic traits relevant to vector control. These assessments included tests of male mating competitiveness against WT mosquitoes, suppression efficacy under varying release ratios, and vector competence for mosquito-borne arboviruses. The results of these experiments provide essential evidence supporting the feasibility of implementing *Wolbachia*-based vector control strategies in Taiwan and represent a critical step toward future field release trials. The development of a locally derived, *Wolbachia*-infected *Ae. aegypti* strain marks a significant milestone in Taiwan’s efforts to adopt sustainable, non-chemical interventions for the control of dengue and other mosquito-borne diseases.

## Materials and methods

### Ethics statement

Mouse blood used in this study was purchased from BioLASCO Taiwan Co., Ltd. (http://www.biolasco.com.tw), a certified commercial supplier. The company operates in full compliance with the Guide for the Care and Use of Laboratory Animals, all relevant national regulations governing the use of live vertebrate animals in research, and the ARRIVE (Animal Research: Reporting of In Vivo Experiments) guidelines. The use of mouse blood (111134_2023.11.24) in this study was approved by the Institutional Animal Care and Use Committee of the National Health Research Institutes (NHRI-IACUC-107054-A).

### Mosquito strains and rearing conditions

WT *Ae. aegypti* and *Ae. albopictus* strains were established from larvae and eggs collected through routine entomological surveillance in Kaohsiung City by the National Mosquito-Borne Diseases Control and Research Center (NHRI, Taiwan). Sampling was conducted in four administrative surveillance districts, with approximately 80 ovitraps deployed across multiple field sites to capture spatially representative mosquito populations. Collected specimens were pooled to establish laboratory colonies, ensuring broad representation of local genetic diversity. Mosquitoes were maintained at 28 ± 1 °C and 70 ± 10% relative humidity under a 12 h light/12 h dark photoperiod. Adults were allowed to mate randomly, and females were fed defibrinated, pathogen-free mouse blood (purchased from BioLASCO Taiwan) using an artificial membrane feeding system (Orinno Technology Pte. Ltd., Singapore). Eggs were hatched after 2 days in dechlorinated water, and larvae were reared in plastic trays and fed a 1:1 mixture of yeast powder (Taiwan Sugar Corp.) and goose liver powder (7573, NTN Corporation, Taiwan). Pupae were transferred to acrylic cages for adult emergence, and adult mosquitoes were provided with a constant supply of 10% sucrose solution.

### Establishment of a Taiwan-derived *w*AlbB-infected *Aedes aegypti* line

A local *w*AlbB-infected *Ae. aegypti* line was established via embryonic microinjection using cytoplasmic extract from wild *Ae. albopictus* eggs collected in Kaohsiung, Taiwan, following protocols modified from Xi et al. [38,39]. Briefly, 4–6-day-old *Ae. albopictus* females collected from the field were dissected under sterile conditions to isolate ovaries. Ovarian tissues were homogenized in phosphate-buffered saline (PBS) and filtered through a 5 µm membrane to obtain a *Wolbachia*-containing cytoplasmic suspension. Freshly laid *Ae. aegypti* embryos (0–1 h post-oviposition) were aligned on coverslips coated with double-sided adhesive tape and overlaid with halocarbon oil to prevent desiccation. Approximately 1–2 nL of the cytoplasmic suspension was injected into the posterior pole of each embryo using a fine glass capillary under a micromanipulator system. Injected embryos were incubated at 25–28 °C under high humidity until hatching. F₀ larvae were reared to adulthood and crossed with WT *Ae. aegypti* to produce F₁ progeny. In each subsequent generation, *w*AlbB-infected individuals were selected and backcrossed with WT *Ae. aegypti* for at least nine generations to ensure genetic background homogenization and stable maternal transmission of *Wolbachia*. At each generation, backcrossing was performed using population-level WT mosquitoes derived from the established field colony, rather than individuals from a single lineage. The WT colony was maintained at a large population size to minimize potential genetic drift during introgression and to preserve the representative local genetic background. Female mosquitoes in each generation were screened for *w*AlbB infection using specific PCR primers (forward: 5′-ACGTTGGTGGTGCAACATTTG-3′; reverse: 5′-TAACGAGCACCAGCATAAAGC-3′). The resulting *w*AlbB-infected *Ae. aegypti* strain (designated *w*AlbB-Tw) consistently maintained an infection rate above 95% and was used for all subsequent experiments.

### Genomic sequencing and comparative analysis of *Wolbachia*

Total DNA was extracted from laboratory colonies of *Ae. aegypti* infected with the *Wolbachia* strain *w*AlbB using a standard phenol–chloroform protocol with RNase treatment. DNA quality and concentration were assessed by fluorometry and agarose gel electrophoresis. Long-read whole-genome sequencing was carried out on the Oxford Nanopore MinION platform with a ligation-based library preparation kit. *De novo* assembly of sequencing reads was performed using standard long-read workflows optimized for bacterial genomes. The assembled contigs were taxonomically classified to confirm their *Wolbachia* origin and annotated with the NCBI Prokaryotic Genome Annotation Pipeline. Comparative analysis with publicly available references indicated that the assembled genome is highly similar to *w*AlbB (accession no. CP031221.1) and clearly distinct from *w*AlbA (accession no. CP101657.1), thereby supporting the identity of the *Wolbachia* strain used in this study.

### Phylogenetic analysis of *Wolbachia* genomes

To determine the evolutionary placement of the transinfected *w*AlbB-Tw-Kao strain, comparative phylogenomic analyses were conducted using representative *Wolbachia* genomes from Supergroups A and B. Complete genome sequences were retrieved from NCBI GenBank, including *w*AlbB reference strains (e.g., CP031221.1 and *w*AlbB-Uju), *w*AlbA (CP101657.1), *w*Mel, *w*Ri, and *w*Pip. All genomes were re-annotated using Prokka v1.14.6 to ensure consistent gene prediction across datasets [40]. Single-copy core genes shared among all genomes were identified using Roary v3.13.0 with a 95% sequence identity threshold [41]. The resulting core gene sequences were concatenated to generate a core-genome alignment. Maximum-likelihood phylogenetic reconstruction was performed using RAxML v8.2.12 under the GTR + Γ nucleotide substitution model with 1,000 bootstrap replicates to assess branch support [42]. The resulting phylogeny placed *w*AlbB-Tw-Kao within the Supergroup B clade, clustering with previously characterized *w*AlbB strains and clearly separated from Supergroup A lineages.

### Quantification of *w*AlbB density by real-time PCR

To enable absolute quantification of *w*AlbB copy number in mosquito samples, we developed a SYBR Green–based real-time PCR assay targeting the CinA gene of the *w*AlbB strain, with absolute copy numbers derived from a plasmid standard curve [43]. Primer sequences were designed based on the *w*AlbB CinA gene and evaluated for specificity by in silico alignment against the *w*AlbA reference genome (CP101657.1), which revealed multiple mismatches in the primer binding regions. A pair of cloning primers (*w*AlbB-CinA Q-PCR fragment cloning F: AGGGAATTGGGAATTCCCGCGGGGAGCAATCTATTTCCTC and R: CCTCGAGCCGCGGCCGCACTTACCCCTTGTCTACTTGGA) was designed to amplify the target region using genomic DNA from *Ae. albopictus* as a template. The resulting amplicon was cloned into the EcoRI and NotI sites of a doubly digested pUAST-attB plasmid using the In-Fusion HD Cloning Kit (Takara Bio, Japan). The recombinant plasmid, designated pUAST-attB_*w*AlbB-CinA, was purified using the Plasmid Midi Kit (Qiagen, Germany) and quantified spectrophotometrically. A ten-fold serial dilution (4 ng to 400 fg) was prepared, corresponding to a theoretical range of 2.2 × 10⁹ to 4.63 × 10⁴ copies per reaction, based on plasmid size and molecular weight. Quantitative PCR reactions were performed in triplicate using the specific primer pair (*w*AlbB-CinA Q-PCR-F: CCGCGGGGAGCAATCTATTT and Q-PCR-R: ACTTACCCCTTGTCTACTTGGA) and the KAPA SYBR FAST qPCR Master Mix (Sigma-Aldrich), on an ABI ViiA7 system. Each 10 μL reaction contained 2 μL of plasmid DNA, 0.125 μM of each primer, and SYBR master mix, following the manufacturer’s protocol. Thermal cycling conditions were: 95 °C for 3 min; 40 cycles of 95 °C for 15 s and 60 °C for 30 s. Melt curve analysis confirmed specificity of amplification. The standard curve was generated by plotting the cycle threshold (Ct) values against the logarithm of the input plasmid copy number. Linear regression analysis yielded a slope of –3.49 and an intercept of 40.359 (R² = 0.9969), indicating high amplification efficiency (E = 93.4%). This equation (Copy number = 10^ [(40.359 – Ct)/3.49]) was subsequently used to determine the absolute copy number of *w*AlbB in biological samples. Samples with inconsistent triplicate results or Ct values exceeding 35 were excluded from analysis.

### Immunofluorescence staining and confocal imaging of *w*AlbB in mosquito tissues

Dissections of *w*AlbB-Tw-Kao male mosquitoes (1 day old; n = 6) and female mosquitoes (18–20 h post-blood feeding; n = 6) were performed under a stereo microscope. The testes and ovaries were carefully isolated and fixed on ice in 4% paraformaldehyde with 1% Triton X-100 in PBS for 2 h, followed by fixation in 4% paraformaldehyde in PBS for 30 min. Tissues were then washed three times with 0.3% PBS–Triton X-100 for permeabilization and subsequently blocked with 5% normal goat serum in 0.3% PBS–Triton X-100 at 4 °C overnight. Tissues were incubated overnight at 4 °C with a rabbit polyclonal anti-*Wolbachia* antibody (1:500; BOSTER, DZ33973–1) [35]. To confirm staining specificity, negative controls were processed in parallel with omission of the primary antibody. No specific fluorescence signal was detected in these controls. The anti-*Wolbachia* antibody recognizes a conserved *Wolbachia* surface protein and has been validated in previous studies for detection of *Wolbachia* in *Aedes* mosquitoes [44]. As the *w*AlbB-Tw-Kao line harbors only the *w*AlbB strain and not *w*AlbA (confirmed by genomic and PCR analyses), cross-reactivity with other *Wolbachia* strains was not applicable in this experimental context. After three washes with 0.3% PBS–Triton X-100 (0.1% PBS–Triton X-100 for midgut samples), tissues were incubated overnight at 4 °C with Alexa Fluor 488–conjugated goat anti-rabbit IgG (1:500 in PBS–Triton X-100), together with DAPI (1:1000) and Phalloidin Alexa Fluor 647 (1:100; Invitrogen, A22283). Samples were mounted on slides with antifade mounting medium (Vectashield, H-1000) and imaged using a Leica TCS SP5 II confocal laser-scanning microscope (Leica Microsystems, Wetzlar, Germany).

### Cytoplasmic Incompatibility (CI) assay

CI levels were assessed using a cage-based cross-mating assay, following the protocol described by Zabalou et al. [45], with modifications. All crosses were conducted at 28 °C under controlled laboratory conditions. The following four mating combinations were tested: WT male × WT female, *w*AlbB-Tw-Kao male × WT female, WT male × *w*AlbB-Tw-Kao female, and *w*AlbB-Tw-Kao male × *w*AlbB-Tw-Kao female. For each mating combination, 20 virgin females (3 days old) and 20 virgin males (3 days old) were introduced into a mating cage, with three biological replicates per cross. After mating, females were blood-fed using an artificial membrane feeding system and allowed to oviposit on moist filter paper. Eggs were matured on the filter paper for 5 days, then submerged in deoxygenated water under vacuum conditions to stimulate hatching. After 3 days, the number of hatched larvae was counted, and the hatch rate was calculated as an indicator of CI expression.

### Virus strains and maintenance

The TW2015 strain of DENV serotype 2 (DENV-2; GenBank accession no. KU365901) and the Zika virus (ZIKV) strain PRVABC59 were obtained from the Taiwan Centers for Disease Control (Taiwan CDC) and propagated in Vero cells. Briefly, Vero cells were seeded in 175 cm² culture flasks and grown at 28 °C to approximately 80% confluency. Cells were then infected at a multiplicity of infection (MOI) of 0.5 for 3 h at 28 °C. Following infection, the inoculum was removed and replaced with fresh culture medium. The cultures were maintained at 28 °C for 5 days to allow virus replication. Supernatants were harvested, clarified by filtration through a 0.22 μm membrane filter, aliquoted, and stored at −80 °C until use. Viral titers were determined by plaque assay on BHK-21 cells.

### Oral viral infection and saliva collection

Fresh mouse blood was centrifuged at 4 °C for 10 min to separate plasma and cellular components. The plasma was heat-inactivated at 55 °C for 1 h to eliminate complement activity that may otherwise interfere with viral infectivity during oral challenge assays [46,47]. The treated plasma and washed blood cells were then recombined. Virus stocks of DENV-2 (TW2015) or ZIKV were diluted in treated mouse blood to a final concentration of 2 × 10⁷ plaque-forming units per milliliter (PFU/mL). Female mosquitoes (5–7 days old) were allowed to feed on virus-infected blood for 30 min using an artificial membrane feeding system. Across independent biological replicates, approximately 60 mosquitoes were offered infectious blood per group, from which ~40 fully engorged females were obtained (average feeding rate ≈ 70%). Only fully engorged mosquitoes were retained for downstream analyses. At 5, 10, and 15 days post-blood meal (PBM), midguts, salivary glands, and saliva were collected from a total of 40 mosquitoes per group. Saliva was harvested following a previously described protocol [48]. Briefly, the wings and legs of each mosquito were removed, and the proboscis was inserted into a 200-μL pipette tip containing 5 μL of fetal bovine serum (FBS). After 30 min, the saliva-containing FBS was mixed with 45 μL of serum-free RPMI medium and subjected to plaque assay to quantify infectious viral particles. Viral infection rates (IR) were defined as the proportion of mosquitoes with detectable infectious virus in the midgut as determined by plaque assay. For viral titer quantification, tissues from infected individuals were analyzed individually using standard plaque assays on BHK-21 cells. Samples with no detectable plaques were recorded as uninfected and excluded from mean viral titer calculations but included in IR analyses. Uninfected blood-fed mosquitoes were included as negative controls to confirm the specificity of viral detection in both midgut and saliva samples.

### Plaque assay

To determine the titers of DENV-2 and ZIKV, BHK-21 cells were seeded at a density of 2 × 10⁵ cells per well in six-well plates and incubated for 24 h at 37 °C in an incubator. Virus-containing supernatants were serially diluted (10 ⁻ ¹ to 10 ⁻ ⁶) in Dulbecco’s Modified Eagle Medium (DMEM) and added to the BHK-21 monolayers. After 2 h of adsorption, the inoculum was removed and the cells were washed with PBS. The infected cells were then overlaid with DMEM supplemented with 1% methyl cellulose and 5% FBS, and incubated for 5 days at 37 °C. Following incubation, the overlay medium was removed, and cells were fixed and stained with 0.5% crystal violet solution [49]. Uninfected midgut and saliva samples from blood-fed mosquitoes were processed in parallel as negative controls to confirm assay specificity and exclude background contamination. Visible plaques were counted, and viral titers were expressed as PFU/mL.

### Insecticide susceptibility bioassay

Mosquito knockdown and mortality rates were quantified using space spray protocols previously described by Gray et al. [50]. Female mosquitoes from three *Ae. aegypti* strains were tested: a WT field-derived strain collected from Kaohsiung, Taiwan; the *Wolbachia*-infected *w*AlbB-Tw-Kao strain; and the laboratory-maintained Liverpool strain known to be insecticide-susceptible. Non-blood-fed female mosquitoes aged 3–5 days were used in all bioassays to minimize physiological variability that may influence insecticide susceptibility. At the time of testing, the WT colony had been maintained at F6–F7 following field establishment, while the *w*AlbB-Tw-Kao line had undergone nine generations of backcrossing with this field-derived WT population and was maintained at F7–F9 prior to experimental evaluation. Three insecticides commonly used in Taiwan were tested at two concentrations each: etofenprox at 4000 ppm and 250 ppm, cypermethrin at 150 ppm and 9.4 ppm, and permethrin at 1 ppm and 0.25 ppm. For each treatment, 25 female *Ae. aegypti* mosquitoes from a single strain were placed in a cylindrical nylon-mesh bioassay cage (approximately 10 × 5 cm) suspended 30 cm above the floor. Prior to exposure, cages were acclimated for 30 min in a sealed room (3.1 × 2.3 × 3 m) with no air circulation. Technicians wearing appropriate personal protective equipment (gloves and masks) applied the insecticide using a horizontal spraying angle for 10s. Each insecticide treatment was replicated three times. Knockdown was assessed by 30 min post-exposure, defined as the inability of a mosquito to fly (including dead individuals). Mortality was recorded 24 h after spraying.

### Female fecundity assessment

To evaluate the effect of *w*AlbB-Tw-Kao infection on female fecundity, 3- to 7-day-old female mosquitoes were used in the assay. Four mating combinations between WT and *w*AlbB-Tw-Kao mosquitoes were tested. Each combination included three independent biological replicates with 20 females per replicate (total n = 60 females per cross). After a 3-day mating period, females were blood-fed on mouse blood using an artificial membrane feeding system. Following blood feeding, non-blood-fed females and all males were removed from the cages. Three days after blood feeding, female mosquitoes were anesthetized on ice for 5 min and then transferred individually into plastic vials for egg collection. Each vial contained 3 mL of water and a piece of filter paper (3 × 2 cm²). After an additional 3 days, the number of eggs laid by each female on the filter paper was counted, followed by assessments of hatching rate and adult emergence. Females that died during the experiment were excluded from the analysis.

### Mating capability assay

To assess the mating competitiveness of *w*AlbB-Tw-Kao males, four crossing combinations were established: (1) female (♀) *w*AlbB-Tw-Kao × male (♂) *w*AlbB-Tw-Kao, (2) ♀*w*AlbB-Tw-Kao × ♂WT, (3) ♀WT × ♂*w*AlbB-Tw-Kao, and (4) ♀WT × ♂WT. For each cross, five 3-day-old males and twenty 3-day-old virgin females were placed in transparent acrylic cages (30 × 30 × 30 cm) under standard insectary conditions. After 2 h of mating opportunity, females were collected, and their spermathecae were dissected under a stereo microscope to determine the presence of sperm as an indicator of successful insemination. The mating success rate was calculated as the proportion of inseminated females relative to the total number dissected. Each experiment was repeated three times.

### Adult mosquito longevity assay

To evaluate adult mosquito longevity, experiments were conducted under both laboratory and semi-field simulated conditions [51]. Twenty virgin male or female WT and *w*AlbB-Tw-Kao mosquitoes (3 days old) were individually introduced into transparent acrylic cages (30 × 30 × 30 cm) and provided with unlimited 10% sucrose solution. In the semi-field setting, environmental conditions, including temperature, humidity, and photoperiod, were governed by natural fluctuations. During the study period (July to September), temperatures ranged from 28 °C to 35 °C, relative humidity fluctuated between 80% and 95%, and the photoperiod was approximately 14 h per day. For both types of experiments, dead mosquitoes were removed and counted daily until no individuals remained alive. Each condition was replicated three times.

### Male mating competitiveness assay

To evaluate the mating competitiveness of *Wolbachia*-infected male *Ae. aegypti* (*w*AlbB-Tw-Kao), we conducted cage experiments using varying ratios of virgin WT females, WT males, and *w*AlbB-Tw males (♂*w*AlbB-Tw-Kao). The experimental ratios of ♀WT: ♂WT: ♂*w*AlbB-Tw-Kao were as follows: 1:1:0, 1:1:1, 1:1:3, 1:1:5, and 1:1:10, respectively. Each condition included 10 WT males and 0, 10, 30, 50, or 100 *w*AlbB-Tw-Kao males, depending on the group, and was replicated five times. Before introducing 10 virgin WT females, WT and *w*AlbB-Tw-Kao males were first introduced together for 2 h in acrylic cages (30 × 30 × 30 cm). Mosquitoes were then allowed to mate freely for 3 days under standard insectary conditions. Females were blood-fed for 30 min, and 2 days later, oviposition cups were placed inside the cages for egg collection. Egg numbers and hatching rates were recorded using collected egg papers. After 5 days of egg maturation on wet filter paper, eggs were immersed in deoxygenated water. Three days later, hatched eggs were counted to determine hatch rates.

### Fried competitiveness index (C) calculation

To quantify the relative mating competitiveness of *w*AlbB-Tw-Kao males under competitive conditions, the Fried competitiveness index (C) was calculated following the method originally described by Fried [52]. This index estimates the mating competitiveness of incompatible (sterile) males relative to WT males based on observed egg hatch rates under mixed mating conditions. For each release ratio, the competitiveness index (C) was calculated using the formula:


$$C=\frac{H_{\text{0}}-H}{H-H_{s}}\times \frac{N}{s}$$

where:

H₀ represents the mean hatch rate in the fully compatible control cross (♀WT × ♂WT), H represents the observed hatch rate in the competitive mating treatment,

Hₛ represents the hatch rate in fully incompatible crosses (assumed to be 0% based on complete cytoplasmic incompatibility),

N is the number of WT males,

and S is the number of *w*AlbB-Tw-Kao males released.

Calculations were performed independently for each biological replicate (n = 4 per ratio), and mean C values ± SD were reported. A C value of 1 indicates equal competitiveness between incompatible and WT males, whereas values <1 indicate reduced competitiveness of *w*AlbB-Tw-Kao males relative to WT males.

### Statistical analysis

All statistical analyses were performed using GraphPad Prism version 8.0 and R software version 4.1.0. Differences in viral titers between WT and *w*AlbB-infected mosquitoes were analyzed using the non-parametric Mann–Whitney U test. Mosquito lifespan data were evaluated using the log-rank (Mantel–Cox) test implemented in R [53]. Mating competitiveness was assessed by comparing egg hatch rates using paired *t*-tests. A *p*-value of less than 0.05 was considered statistically significant for all tests.

## Results

### Generation and confirmation of *w*AlbB-transinfected *Aedes aegypti* line

To establish a Taiwan-derived *Ae. aegypti* line stably infected with *Wolbachia*, cytoplasm extracted from eggs of field-collected *Ae. albopictus* in Kaohsiung was microinjected into preblastoderm-stage *Ae. aegypti* embryos. Of the 1,120 embryos injected, 127 (11.3%) successfully hatched into F₀ larvae, of which 29 (2.6%) developed into adult females. Following oviposition, individual F₀ females were screened by PCR using both *w*AlbA- and *w*AlbB-specific primers. Two F₀ females were confirmed to be *w*AlbB-positive and *w*AlbA-negative, indicating successful transinfection without co-transfer of *w*AlbA (S1A Fig). These independent infected founder females were subsequently used to initiate the transinfected line. To assess infection stability, the presence of *w*AlbB was monitored across nine consecutive generations. Quantitative PCR analysis confirmed that 100% of offspring remained *w*AlbB-positive in each generation (S1B Fig), demonstrating complete maternal transmission and stable establishment of the *w*AlbB-Tw-Kao line.

**Fig 1:**
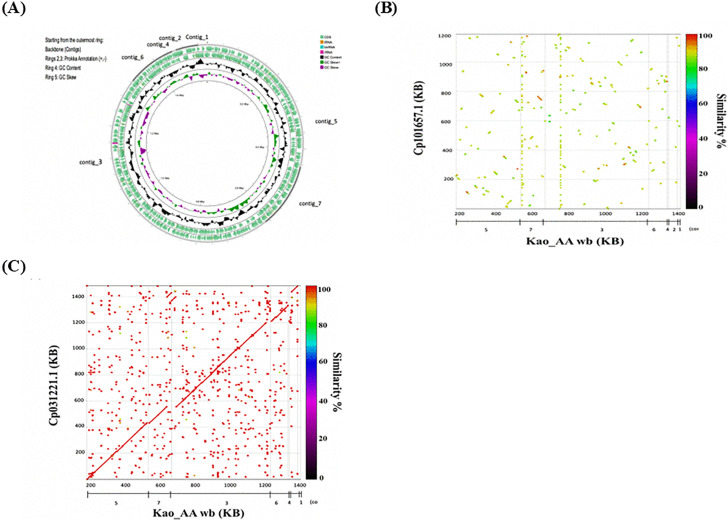
Genomic characterization of *Wolbachia* from transinfected *Ae. aegypti.* **(A)** Circular representation of the de novo–assembled *Wolbachia* genome from transinfected *Ae. aegypti*. The genome consists of seven contigs, which were ordered and oriented based on conserved synteny with the *w*AlbB reference genome (accession no. CP031221.1) to facilitate visualization. Displayed features include predicted coding sequences (CDSs), tRNAs, rRNA operons, and guanine–cytosine (GC) content. **(B)** Dot plot comparison with *w*AlbA revealed minimal similarity. **(C)** Dot plot comparison with *w*AlbB showed strong collinearity, indicating close relatedness.

To provide genome-level support for the identity of the *Wolbachia* strain introduced into the local *Ae*. *aegypti* line, we performed whole-genome sequencing on total genomic DNA extracted from infected mosquitoes. Assembly of the sequencing data yielded a *Wolbachia* draft genome comprising seven contigs (total approximately 1.6 Mb; GC 34.4%). These *Wolbachia* contigs showed strong collinearity with the reference *w*AlbB genome (CP031221.1) and only sparse similarity to *w*AlbA (CP101657.1) (Fig 1). BUSCO analysis indicated that over 96% of the expected single copy orthologs were recovered, supporting a high level of genome completeness. To further resolve its evolutionary placement and assess relatedness to previously described *w*AlbB variants, we conducted comparative genomic analyses including representative *Wolbachia* genomes from Supergroups A and B. Maximum-likelihood phylogenetic reconstruction based on concatenated single-copy core genes placed the *w*AlbB-Tw-Kao genome within the Supergroup B clade, clustering with characterized *w*AlbB strains (S2 Fig), and clearly separated it from Supergroup A lineages. Together, these findings confirm that the *Wolbachia* in the infected line corresponds to *w*AlbB, consistent with its origin from local *Ae*. *albopictus*.

**Fig 2:**
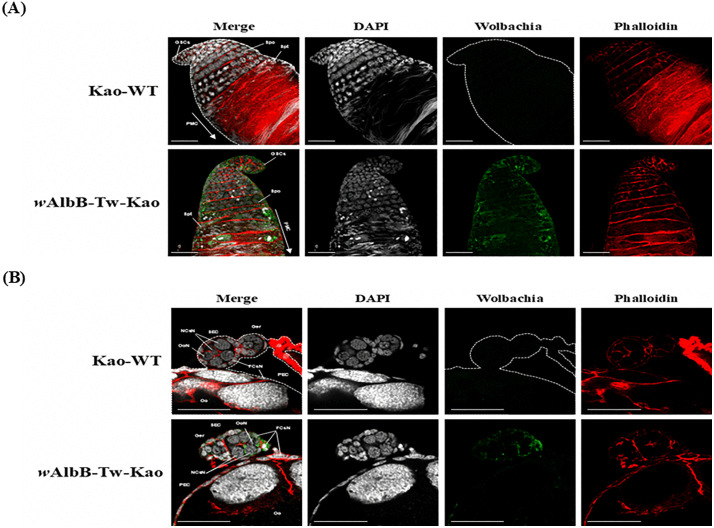
Presence of *Wolbachia* in the testes and ovaries of *w*AlbB-Tw-Kao *Ae. aegypti* line. Immunofluorescence staining revealed the distribution of *Wolbachia* (green) in transinfected *Ae. aegypti* **(A)** testes and **(B)** ovaries. Testes and ovaries from six independent individuals per sex were analyzed across two independent experimental replicates. DNA was counterstained with DAPI (white), and the cytoskeleton was labeled with phalloidin (red). GSCs: germline stem cells, Spc: spermatocyte, Spt: spermatid, PMC: post-meiosis cell, Ger: germarium, Oo: oocyte, OoN: oocyte nucleus, NCsN: nurse cell nucleus, FCsN: follicle cell nucleus, PEC: primary egg chamber, SEC: secondary egg chamber. Scale bar: 50 μm. WT: wild-type local strain.

In addition, the presence of *w*AlbB in transinfected mosquitoes was further validated by immunofluorescence assays. As shown in Fig 2, *Wolbachia* signals were detected in the testis and ovaries of the *w*AlbB-infected local *Ae. aegypti* line. Collectively, these findings confirm the successful establishment of the transinfected line. The resulting *Wolbachia*-infected Taiwanese *Ae. aegypti* strain was designated as “*w*AlbB-Tw-Kao” and used in subsequent experiments.

### Assessment of CI induced by the *w*AlbB-Tw-Kao line

**Fig 3:**
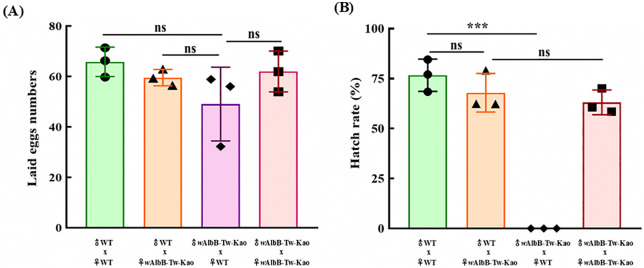
Cytoplasmic incompatibility (CI) analysis in the *w*AlbB-Tw-Kao *Ae. aegypti* line. **(A)** Number of eggs laid and **(B)** egg hatch rate from reciprocal crosses between wild-type (WT) and *w*AlbB-Tw-Kao mosquitoes. Mating combinations included ♂WT × ♀WT, ♂WT × ♀*w*AlbB-Tw-Kao, ♂*w*AlbB-Tw-Kao × ♀WT, and ♂*w*AlbB-Tw-Kao × ♀*w*AlbB-Tw-Kao. Each data point represents the result from one independent biological replicate (n = 3), with 20 males and 20 females per mating combination in each replicate. Bars indicate the mean across experiments, and error bars represent the standard deviation. Statistical significance was determined using Student’s t-test. Exact *P* values are reported in the Results section.***p < 0.001; ns, not significant. WT: local wild-type strain from Kaohsiung.

To further characterize the effect of CI induced by the *w*AlbB-Tw-Kao strain, mating experiments were conducted in transparent acrylic cages. In all experimental groups, females successfully laid eggs (Fig 3A). However, in the test group consisting of 20 uninfected WT females crossed with 20 *w*AlbB-Tw-Kao males, the egg hatch rate was 0% (Fig 3B), indicating complete CI. In contrast, the reciprocal cross between *w*AlbB-Tw-Kao females and WT males yielded a hatch rate of 67.9 ± 6.8%. Control crosses between WT males and females and between *w*AlbB-Tw-Kao males and females produced hatch rates of 77.5 ± 3.1% and 63.1 ± 4.4%, respectively. These findings confirm that *w*AlbB-Tw-Kao males induce strong CI when mating with uninfected WT females.

### Reduced replication of DENV and ZIKV in *w*AlbB-Tw-Kao *Aedes aegypti* line

To further assess whether the newly generated *w*AlbB-Tw-Kao mosquitoes exhibit viral suppression, virus inhibition assays were performed. Female mosquitoes were orally infected with either DENV-2 or ZIKV. At 5-, 10-, and 15-days PBM, midguts, salivary glands, and saliva were collected to evaluate viral titers, infection rates (IR), and transmission rates (TR). Viral titers were quantified using plaque assays.

**Fig 4:**
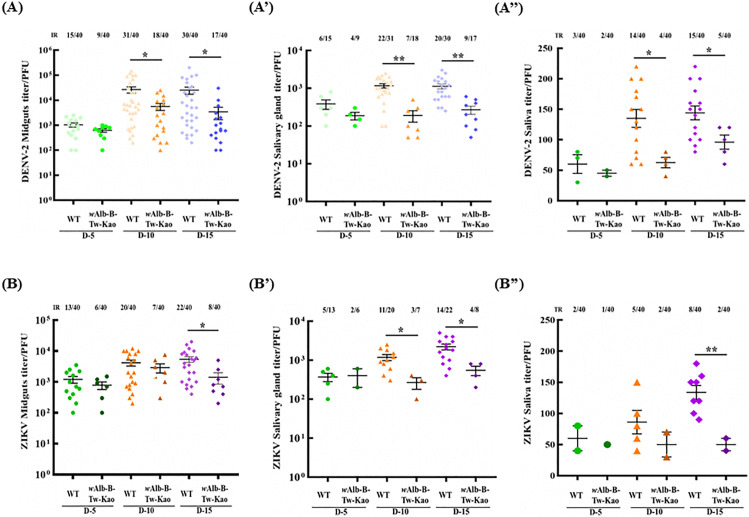
Inhibition of dengue virus (DENV-2) and Zika virus (ZIKV) infection by the *w*AlbB-Tw-Kao mosquito line. *Wolbachia*-transinfected *Ae. aegypti* (*w*AlbB-Tw-Kao) and Kaohsiung local wild-type (WT) mosquitoes were challenged with **(A–A”)** dengue virus serotype 2 (DENV-2, TW2015) or **(B–B”)** ZIKV. Midguts, salivary glands, and saliva were collected at 5, 10, and 15 days post-blood meal (PBM). Viral titers were quantified by plaque assay on BHK-21 cells. For each group (n = 40 mosquitoes), viral titers are shown as mean ± SD. Samples negative for virus detection were excluded from titer calculations. Infection rates (IR) are presented as the percentage of infected individuals (positive/tested) and indicated above each panel. Transmission rate (TR) was defined as the proportion of saliva-positive individuals among tested mosquitoes. Differences in viral titers between groups were analyzed using the Mann–Whitney rank-sum test. Exact *P* values are reported in the Results section. Statistical significance is indicated as follows: *p < 0.05; **p < 0.01.

Following oral infection with DENV-2 at a concentration of 2 × 10⁷ PFU/mL, the infection rate (IR) in *w*AlbB-Tw-Kao mosquitoes was reduced relative to WT at 5 days PBM (22.5% vs. 37.5%), with similar reductions observed at 10 and 15 days PBM (D10: 45.0% vs. 77.5%; D15: 42.5% vs. 75.0%; Fig 4A). These mosquitoes were also less capable of transmitting DENV-2, exhibiting lower transmission rates (TR) at 10 days PBM (WT: 35.0 ± 1.4%; *w*AlbB-Tw-Kao: 10.0 ± 2.5%; Fig 4A”) and at 15 days PBM (WT: 37.5 ± 4.0%; *w*AlbB-Tw-Kao: 12.5 ± 4.8%; Fig 4A”). Consistent with these reductions in IR and TR, *w*AlbB-Tw-Kao females exhibited a marked decrease in viral replication. At 10 days PBM, the median DENV-2 titer in the midgut was significantly lower in *w*AlbB-Tw-Kao mosquitoes than in WT controls (5.6 × 10³ vs. 2.6 × 10⁴ PFU/mL; *p* = 0.039, Mann–Whitney test). This reduction persisted through 15 days PBM (3.4 × 10³ vs. 2.5 × 10⁴ PFU/mL; *p* = 0.048). Comparable reductions were observed in salivary glands at 10 days PBM (190.5 vs. 1.2 × 10³ PFU/mL; *p* = 0.001) and 15 days PBM (270 vs. 1.2 × 10³ PFU/mL; *p* = 0.003). Low viral titers were also detected in the saliva of *w*AlbB-Tw-Kao mosquitoes at both 10 and 15 days PBM compared with WT counterparts (D10: 62.5 vs. 135.3 PFU/mL, *p* = 0.022; D15: 96.5 vs. 144 PFU/mL, *p* = 0.035; Fig 4A”).

In the ZIKV inhibition assay, reductions in IR and TR were similarly observed in *w*AlbB-Tw-Kao mosquitoes (Fig 4B–4B”). Although no significant reduction in viral titers was observed at 5 or 10 days PBM, a decrease in ZIKV titer was detected in the midguts of *w*AlbB-Tw-Kao females at 15 days PBM (1.4 × 10³ vs. 5.4 × 10³ PFU/mL; *p* = 0.03; Fig 4B). Reduced viral titers were also observed in the salivary glands (D10: 266.6 vs. 1.2 × 10³ PFU/mL, *p* = 0.049; D15: 550.5 vs. 2.2 × 10³ PFU/mL, *p* = 0.038; Fig 4B’) and saliva (50.0 vs. 133.8 PFU/mL, *p* = 0.001; Fig 4B”). These reductions in IR and TR were similar to those observed for DENV-2.

### Insecticide susceptibility of *w*AlbB-Tw-Kao mosquitoes

**Fig 5:**
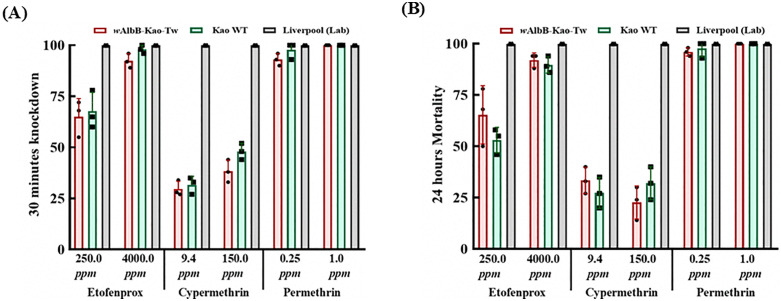
Susceptibility of *Ae. aegypti* line to three commonly used insecticides in space spray assays. Female mosquitoes from three strains—the Kaohsiung local wild-type strain (WT), the *Wolbachia*-infected *w*AlbB-Tw-Kao line, and the insecticide-susceptible Liverpool laboratory strain (Lab)—were tested to evaluate knockdown and mortality responses. **(A)** Knockdown was assessed 30 min post-exposure and defined as the inability to fly, including both moribund and dead individuals. **(B)** Mortality was recorded 24 h after spraying. Each treatment consisted of three independent biological replicates, with 25 females per replicate. Data are presented as mean ± standard deviation (SD).

To evaluate insecticide susceptibility in the newly established *Wolbachia*-infected line, we compared the *w*AlbB-Tw-Kao strain with the uninfected local WT strain across several pyrethroid compounds, including etofenprox, cypermethrin, and permethrin (Fig 5). In assays with etofenprox, both strains demonstrated moderate susceptibility at low concentrations, with comparable knockdown rates (65.0 ± 10.8% vs. 68.3 ± 11.8%) and mortality rates (65.3 ± 9.4% vs. 53.3 ± 2.4%) for *w*AlbB-Tw-Kao and WT, respectively. At higher concentrations, both groups exhibited complete susceptibility, with nearly 100% knockdown and mortality (*p* > 0.05, Mann–Whitney test). Both *w*AlbB-Tw-Kao and WT *Ae. aegypti* exhibited notable resistance to cypermethrin at both low (9.4 ppm) and high (150 ppm) concentrations, as indicated by reduced knockdown and mortality rates. For permethrin, both strains were highly susceptible regardless of concentration. In contrast, the laboratory reference Liverpool strain exhibited complete susceptibility (100% knockdown and mortality) across all tested insecticides and concentrations. Overall, no significant differences in insecticide susceptibility were detected between the *w*AlbB-Tw-Kao and WT strains across all tested compounds and concentrations (all comparisons, *p* > 0.05), indicating comparable resistance profiles.

### Mosquito fitness and mating behavior

**Fig 6:**
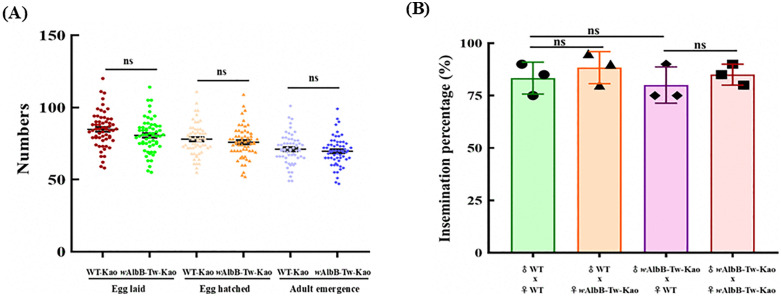
Reproductive fitness and insemination success of the *w*AlbB-Tw-Kao *Ae. aegypti* line. **(A)** Reproductive output of *w*AlbB-Tw-Kao females (F8 ~ F9) compared with wild-type (WT) females from a local *Ae. aegypti* field strain in Kaohsiung, measured by the number of eggs laid, eggs hatched, and adult emergence. **(B)** Insemination success of *w*AlbB-Tw-Kao males assessed across four mating combinations with WT and *w*AlbB-Tw-Kao females under non-competitive conditions. Successful insemination was determined by dissecting the spermathecae of female mosquitoes under a stereomicroscope to confirm the presence of sperm. For both (A) and (B), data represent three independent biological replicates with 20 females per replicate (total n = 60 per group) and are presented as mean ± standard deviation (SD). Statistical significance was evaluated using the Mann–Whitney test (A) and Fisher’s exact test (B). ns, not significant.

For *Wolbachia*-based mosquito release strategies, both fitness and mating performance are key considerations. We assessed the fitness of the *w*AlbB-Tw-Kao strain using the Mann–Whitney U test, with reproductive parameters including the number of eggs laid, number of hatched larvae, and number of surviving adults, and compared these with those of the WT strain. WT females laid slightly more eggs than *w*AlbB-Tw-Kao females (84.7 ± 4.2 vs. 80.5 ± 2.2; *p* = 0.15; Fig 6A), while no significant differences were observed in the number of hatched larvae (78.7 ± 2.1 vs. 75.9 ± 2.1; *p* = 0.29) or adult emergence rates (71.1 ± 1.4 vs. 69.5 ± 1.9; *p* = 0.44). These results indicate that *w*AlbB-Tw females maintain normal reproductive capacity comparable to that of the WT strain.

To further evaluate whether *Wolbachia* infection affects male insemination success, we mated *w*AlbB-Tw-Kao males with virgin females and used the presence of sperm in the spermathecae as an indicator of successful insemination (Fig 6B). Across four mating combinations, insemination rates ranged from 80% to 88%, with no significant differences compared with the WT control cross (83.3 ± 5.1%; *p* > 0.05). These findings suggest that *w*AlbB-Tw-Kao males exhibit insemination rates comparable to those of WT males under non-competitive conditions.

### Mosquito longevity assessment

The lifespan of *Wolbachia*-infected mosquitoes is a critical factor in determining release frequency for population suppression strategies. To assess this, we compared the longevity of male and female *w*AlbB-Tw-Kao mosquitoes with that of WT *Ae. aegypti*. Each longevity assay was initiated with 20 adult mosquitoes and conducted under controlled laboratory conditions (S3A Fig). Based on log-rank tests, no significant differences in lifespan were observed between *w*AlbB-Tw-Kao and WT strains for males (*p* = 0.62, 0.49, and 0.45; three replicates) or females (*p* = 0.33, 0.72, and 0.44; three replicates).

To evaluate mosquito survival under more naturalistic conditions, additional longevity experiments were conducted in semi-field enclosures (S3B Fig). Statistical analyses revealed no significant differences in male survival between WT and *w*AlbB-Tw-Kao strains (*p* = 0.28, 0.38, and 0.66; three replicates, log-rank test). Similarly, no significant differences were detected in female survival (*p* = 0.28, 0.52, and 0.44). These findings indicate that *Wolbachia* infection does not negatively affect the lifespan of *Ae. aegypti* males or females.

### Mating competitiveness of *w*AlbB-Tw-Kao males

**Fig 7:**
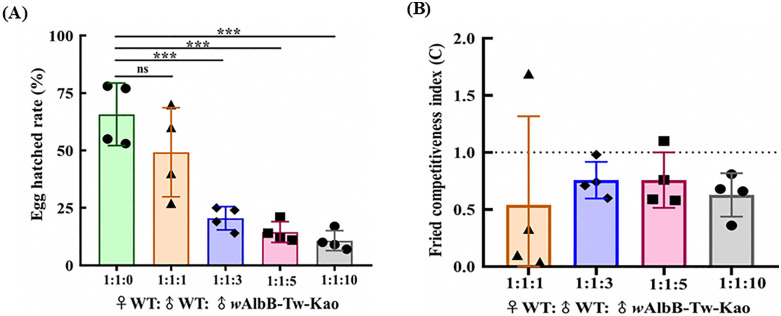
Mating competitiveness of *w*AlbB-Tw-Kao males under laboratory cage conditions. Laboratory cage experiments were conducted with fixed numbers of uninfected females (♀WT) and uninfected males (♂WT), with increasing numbers of *w*AlbB-Tw-Kao males (♂*w*AlbB-Tw-Kao) to generate the indicated release ratios. **(A)** Egg hatch rates (%) observed under each mating ratio. Values represent mean ± SD from four independent biological replicates (n = 4; 10 females per replicate). **(B)** Fried competitiveness index (C) calculated for each release ratio. The dashed horizontal line indicates C = 1 (equal competitiveness between *w*AlbB-Tw-Kao and WT males). Values are shown as mean ± SD across replicates. Exact *P* values are reported in the Results section. Statistical significance is indicated by asterisks: ***p < 0.001.

To quantify the relative mating performance of *w*AlbB-Tw-Kao males under competitive conditions, laboratory cage assays were conducted using varying release ratios of uninfected females (♀WT), uninfected males (♂WT), and *w*AlbB-Tw-Kao males (♂*w*AlbB-Tw-Kao). Hatch rates declined in a ratio-dependent manner as the proportion of *w*AlbB-Tw-Kao males increased (Fig 7A). Mean egg hatch rates (± SD) across four biological replicates were 65.7 ± 11.6%, 49.3 ± 16.6%, 20.9 ± 5.8%, 14.6 ± 5.5%, and 11.2 ± 4.2% for release ratios of 1:1:0, 1:1:1, 1:1:3, 1:1:5, and 1:1:10, respectively. Compared with the fully compatible control (1:1:0), hatch rates were significantly reduced at release ratios of 1:1:3, 1:1:5, and 1:1:10 (all *p* < 0.001), whereas the reduction at 1:1:1 was not significant. To estimate male mating competitiveness, the Fried competitiveness index (C) was calculated for each release ratio, assuming complete incompatibility (0% hatch) in incompatible matings (Fig 7B). Across overflooding ratios of 1:1:3, 1:1:5, and 1:1:10, mean C values ranged from 0.58 to 0.76, indicating that *w*AlbB-Tw-Kao males exhibited approximately 60–75% of the mating competitiveness of WT males under laboratory conditions. Greater variability was observed at the 1:1:1 ratio, likely reflecting stochastic mating dynamics at lower release ratios. Collectively, these results demonstrate that *w*AlbB-Tw-Kao males induce strong cytoplasmic incompatibility under competitive mating conditions, although their relative mating competitiveness is moderately reduced compared to WT males.

## Discussion

Dengue remains a major public health challenge in Taiwan, where *Ae. aegypti* mosquitoes are the main vectors. A promising strategy to control dengue involves the use of *Wolbachia*, a naturally occurring bacterium that can be deployed either to suppress mosquito populations through cytoplasmic incompatibility or to reduce viral transmission via pathogen interference. In this study, we created a locally derived *Ae. aegypti* strain (*w*AlbB-Tw-Kao) carrying the *w*AlbB strain of *Wolbachia* derived from wild *Ae. albopictus* in Kaohsiung, Taiwan. The strain exhibited complete CI, indicating that *w*AlbB-Tw-Kao males can substantially reduce reproductive output when mated with uninfected females. Moreover, females harboring *Wolbachia* showed markedly reduced DENV transmission potential. Laboratory cage releases of *w*AlbB-Tw-Kao males achieved up to 90% suppression of WT mosquito populations. Importantly, our cage experiments were designed to quantify CI-mediated suppression and male mating competitiveness rather than multi-generation replacement dynamics. Nevertheless, the combination of stable maternal transmission and robust virus blocking observed in *w*AlbB-Tw-Kao represents key biological prerequisites for population replacement and supports future evaluation in dedicated multi-generation cage or semi-field studies. Collectively, these findings indicate that the *w*AlbB-Tw-Kao line possesses functional characteristics compatible with both suppression and replacement frameworks and warrants further translational assessment under field-relevant conditions in Taiwan.

Beyond PCR-based screening, we carried out genome sequencing to confirm the identity of the *Wolbachia* strain in the *Ae. aegypti* line (*w*AlbB-Tw-Kao). Although the *Wolbachia* genome was not completely assembled, the draft contigs covered the vast majority of the *w*AlbB reference genome and displayed strong synteny. BUSCO analysis indicated that nearly all expected single copy orthologs were present, supporting a high level of genome completeness (Fig 1). Together, these findings demonstrate that the *Wolbachia* strain in the infected line corresponds to *w*AlbB, consistent with its origin from *Ae. albopictus.*

Immunofluorescence assays further confirmed the presence of *w*AlbB in both the testis and ovaries of infected mosquitoes, indicating the successful establishment of the infection line (Fig 2). Its localization within reproductive tissues underscores the capacity of the constructed mosquito line for stable maternal vertical transmission and the induction of CI, both of which are fundamental mechanisms underlying replacement or suppression strategies. Moreover, *w*AlbB infection exerted only minor fitness effects on the locally derived *Ae. aegypti* line, particularly with respect to fecundity, egg hatch rate, and survival (Figs 6 and S3). These traits are particularly relevant for field application, as the persistence and spread of *Wolbachia*-infected mosquitoes depend not only on CI and virus blocking but also on maintaining survival and reproductive competitiveness comparable to WT populations [54–56]. These findings support the feasibility of *Wolbachia*-based interventions in Taiwan while underscoring the need for further evaluation across diverse host genetic backgrounds and environmental conditions to assess the generality of these outcomes and to optimize strategies for field deployment.

Our fitness and mating performance results for the *w*AlbB-Tw-Kao line are broadly consistent with previous studies of *w*AlbB-infected *Ae. aegypti* established in diverse genetic backgrounds. Liang et al. [29] reported long-term stability of *w*AlbB, characterized by persistent cytoplasmic incompatibility and complete maternal transmission, while noting that fitness and virus-blocking phenotypes can vary depending on host genetic background. Hugo et al. [28] showed that an Australian background *w*AlbB line retained normal reproductive performance and exhibited strong inhibition of dengue and Zika viruses. Similarly, Al-Amin et al. [57] demonstrated minimal fitness costs, complete cytoplasmic incompatibility, and effective dengue suppression in a Bangladesh genetic background. In comparison, the *w*AlbB-Tw-Kao line maintained reproductive output and adult emergence comparable to the local WT population, and males exhibited normal mating competitiveness, while replication of both dengue and Zika viruses was significantly reduced. Collectively, these cross-population comparisons suggest that although quantitative fitness effects and antiviral efficacy may vary among mosquito backgrounds, *w*AlbB consistently confers cytoplasmic incompatibility and viral interference without compromising key traits relevant to both population suppression and replacement strategies.

Notably, the complete cytoplasmic incompatibility observed in the *w*AlbB-Tw-Kao line (0% egg hatch in incompatible crosses) is consistent with previous reports of *w*AlbB-transinfected *Ae. aegypti* lines established in diverse geographic backgrounds, including Malaysia, Australia, China and Bangladesh, where near-complete or complete CI has been documented. Similarly, the magnitude of viral suppression observed in this study—particularly the significant reductions in DENV titers in midguts, salivary glands, and saliva, together with decreased infection and transmission rates—is comparable to antiviral effects reported for other *w*AlbB-infected lines. These cross-study comparisons suggest that the Taiwan-derived *w*AlbB-Tw-Kao line exhibits phenotypic characteristics within the established performance range of *w*AlbB-based interventions, further supporting its translational relevance. Although quantitative differences in CI intensity or virus blocking may arise due to host genetic background or environmental conditions, the core functional traits of complete CI, stable maternal transmission, and broad antiviral activity appear to be conserved across *w*AlbB deployments.

Recent field evidence further supports the translational relevance of *w*AlbB-based *Wolbachia* strategies across diverse epidemiological settings. In Malaysia, large-scale deployments of *w*AlbB-infected *Ae. aegypti* for population replacement were associated with significant reductions in dengue incidence as *Wolbachia* frequencies increased toward fixation [34], highlighting the suitability of *w*AlbB for hot tropical environments. Complementary population suppression approaches using releases of *w*AlbB-infected males under the incompatible insect technique (IIT) or IIT–sterile insect technique (SIT) frameworks have also demonstrated substantial reductions in *Ae. aegypti* abundance in operational trials conducted in Singapore, as well as in urban field applications in China and pilot programs in the United States. Together, these studies demonstrate that robust cytoplasmic incompatibility, male mating competitiveness, and stable *Wolbachia* maintenance are critical determinants of field performance. In this context, the stable maternal transmission and multi-generation persistence of *w*AlbB observed in our Taiwan-derived line provide an essential biological foundation for future regional implementation of both population replacement and suppression strategies.

In our findings, we introduced a Taiwan-derived *w*AlbB strain into the local *Ae. aegypti* genetic background and demonstrated complete cytoplasmic incompatibility together with significant reductions in DENV and ZIKV infection and transmission under laboratory conditions (Figs 3 and 4). Compared with WT mosquitoes, *w*AlbB-Tw-Kao exhibited markedly lower infection and transmission rates, and disseminated infections were rare. These findings indicate that the *w*AlbB-Tw-Kao line retains key functional traits required for *Wolbachia*-based population suppression and virus interference. Further field evaluation will be necessary to determine its capacity for establishment, long-term stability, and sustained antiviral effects in natural populations.

*Wolbachia*-based vector control represents a promising complementary approach for arbovirus mitigation. Successful implementation depends primarily on compatibility between the released mosquito genetic background and local environmental conditions to ensure competitive performance. Although different *Wolbachia* strains may vary in phenotypic traits such as thermal tolerance, there is currently no evidence that locally adapted *Wolbachia* strains per se improve field performance. Long-term field studies have demonstrated stable maintenance of *Wolbachia* infection, associated phenotypes, and genomic integrity across diverse ecological settings [58,29]. Nevertheless, continued monitoring remains important to assess environmental influences, viral evolution, and population dynamics under local conditions.

To optimize locally adapted strains and ensure their survival and reproductive advantage, mosquitoes can be subjected to multigenerational selection under laboratory conditions that simulate local environments (e.g., temperature and humidity), thereby favoring individuals with higher survival, faster development, and greater fecundity. Through genetic background introgression, *Wolbachia*-infected lines may be crossed with diverse local mosquito populations to enhance environmental tolerance and mitigate the limitations of single-genotype adaptation [59,60]. Continuous monitoring of maternal transmission fidelity and infection stability, followed by semi-field evaluations of population competitiveness, is essential. Integrating these approaches could facilitate the establishment of stable and efficient *Wolbachia*-infected populations under natural conditions, thereby realizing their public health benefits.

In conclusion, the Taiwan-derived *w*AlbB-Tw-Kao *Ae. aegypti* line exhibits complete cytoplasmic incompatibility, robust maternal transmission, and effective suppression of dengue virus. Releases of male mosquitoes resulted in a reduction of wild-type populations, supporting the potential of this approach for vector control. In Taiwan’s subtropical climate, where mosquito-borne diseases persist year-round, conventional insecticide-based strategies face increasing limitations due to resistance development and environmental concerns. *Wolbachia*-based interventions therefore represent a sustainable, targeted, and operationally feasible strategy for either mosquito population suppression or transmission interruption through population replacement, depending on the release design.

Several limitations should be acknowledged. Early generations were not explicitly screened for the presence of the *w*AlbA strain, as the primary screening strategy focused on confirming successful establishment of *w*AlbB. However, subsequent strain-specific PCR and whole-genome sequencing consistently detected only *w*AlbB, and no evidence of *w*AlbA was observed in later generations. The line has maintained stable *w*AlbB infection with complete maternal transmission across multiple generations, suggesting that persistent *w*AlbA co-infection is unlikely. Future line establishment and quality control workflows will incorporate routine dual-strain screening to further strengthen strain fidelity and biosafety. Collectively, these findings provide a practical framework for future field deployment and integrated vector management in Taiwan and support the potential of *Wolbachia*-based strategies to achieve sustained public health impact.

## Supporting information

S1 Fig Confirmation and quantification of *w*AlbB-Tw-Kao infection. **(A)** Strain-specific PCR analysis to confirm the presence of *w*AlbB. DNA quality was validated by amplification of the host reference gene RPS7. (B) Quantification of *w*AlbB density by real-time PCR. Ten male and ten female mosquitoes were collected every three generations for analysis. Mkr: DNA 1kb ladder marker, 1: *Aedes aegypti*, 2: *Aedes albopictus*, 3: *w*AlbB-Tw-Kao *Ae. aegypti* line.
(TIF)

S2 Fig Phylogenetic placement of the *w*AlbB-Tw-Kao genome among representative *Wolbachia* strains. A maximum-likelihood tree was constructed based on whole-genome alignments of representative Supergroup A and B genomes. The *w*AlbB-Tw-Kao genome clusters within the canonical *w*AlbB lineage of Supergroup B alongside previously characterized *w*AlbB strains and does not group with *w*AlbA or other *Wolbachia* supergroups.
(TIF)

S3 Fig Comparison of lifespan. The lifespan of male and female mosquitoes from the *w*AlbB-Tw-Kao and WT strains was evaluated under **(A)** laboratory conditions and **(B)** semi-field simulation environments. Each test began with 20 mosquitoes, provided with 10% sucrose solution as a daily food source. Deceased individuals were recorded and removed daily. Lifespan differences were analyzed using the log-rank (Mantel–Cox) test. Data are presented as mean ± standard deviation (SD) from three independent experiments, each comprising three replicates (3 × 20 mosquitoes).
(TIF)
